# Monocyte Transcriptional Profiling Highlights a Shift in Immune Signatures Over the Course of Illness in Schizophrenia

**DOI:** 10.3389/fpsyt.2021.649494

**Published:** 2021-05-14

**Authors:** Jennifer K. Melbourne, Cherise Rosen, Kayla A. Chase, Benjamin Feiner, Rajiv P. Sharma

**Affiliations:** Department of Psychiatry, The Psychiatric Institute, University of Illinois at Chicago, Chicago, IL, United States

**Keywords:** schizophrenia, psychosis, immune, monocyte, transcriptomics, RNAseq, interferon, inflammation

## Abstract

With advanced understanding of the intricate interplay between the immune and central nervous systems in neurological and neuropsychiatric illness, there is renewed interest in the potential contribution of immune dysregulation to the development and progression of schizophrenia. To inform this line of inquiry requires a more nuanced understanding of specific immune changes throughout the course of illness. Here, we utilized a genome-wide sequencing approach to transcriptionally profile circulating monocytes in participants with chronic schizophrenia. These myeloid cells, isolated from whole blood samples, are highly plastic with potentially important disease-modifying functions. Differential gene expression and gene set enrichment analyses, focusing on established monocyte phenotypic signatures, including those related to proinflammatory (“M1-like”) and protective or tissue remodeling (“M2-like”) functions, were carried out. We demonstrate an overall enrichment of both “M1-like” (interferon-alpha, interferon-gamma, lipopolysaccharide acute) and “M2-like” (endotoxin tolerance, glucocorticoid acute) monocyte signatures in the participants with schizophrenia compared to non-psychiatric controls. There was no enrichment of the “M1-like” chronic stress signature or the “M2-like” interleukin-4 signature. Using the Molecular Signatures Database Hallmark gene sets list, the “interferon response” was most strongly enriched in schizophrenia compared to controls. Additionally, an exploratory subgroup analysis based on illness duration suggests a shift in monocyte phenotype with illness progression. Specifically, the “M1-like” interferon-gamma signature shows decreased enrichment accompanied by increased enrichment of opposing “M2-like” signatures in participants with a medium illness duration shifting to a strong enrichment of interferon response signatures only in participants with a long illness duration. These findings related to circulating immune cell phenotype have potentially important implications for understanding the role of immune dysregulation in schizophrenia and are a critical consideration for future study design and immune-targeting treatment strategies.

## Introduction

There is increased interest in the role of the immune system in the development and progression of neuropsychiatric disorders such as schizophrenia. Findings that demonstrate altered immune activity in those with a schizophrenia diagnosis are prevalent, and genome-wide associations studies and epidemiological data further implicate the immune system (1, 2). Recent research highlights the importance of peripherally derived immune cells in mediating immune-brain communication, particularly at the parenchymal borders (3–5). Here, these immune cells and the effectors they secrete have been shown to influence a wide range of processes including learning and memory, social behavior, and mood, under both normal physiological as well as pathological conditions (6, 7). Myeloid cells such as monocytes and macrophages in particular are the subject of intense investigation as they have the capacity to drive inflammation and cause tissue damage, mediate tissue repair and secrete factors that directly or indirectly influence synaptic transmission (8, 9). For example, it has been demonstrated in rodents that following immune activation inflammatory monocytes are recruited to brain borders such as the choroid plexus, and that monocytes can drive both viral-induced learning and memory deficits and stress-induced behavioral alterations (10–12). Importantly, results from clinical trials indicate that adjunct treatment with immune modifying drugs may improve symptoms in schizophrenia and thus understanding the nature of alterations to immune activity in relation to illness progression may advance the development of new treatment targets (13–16).

**Figure 1:**
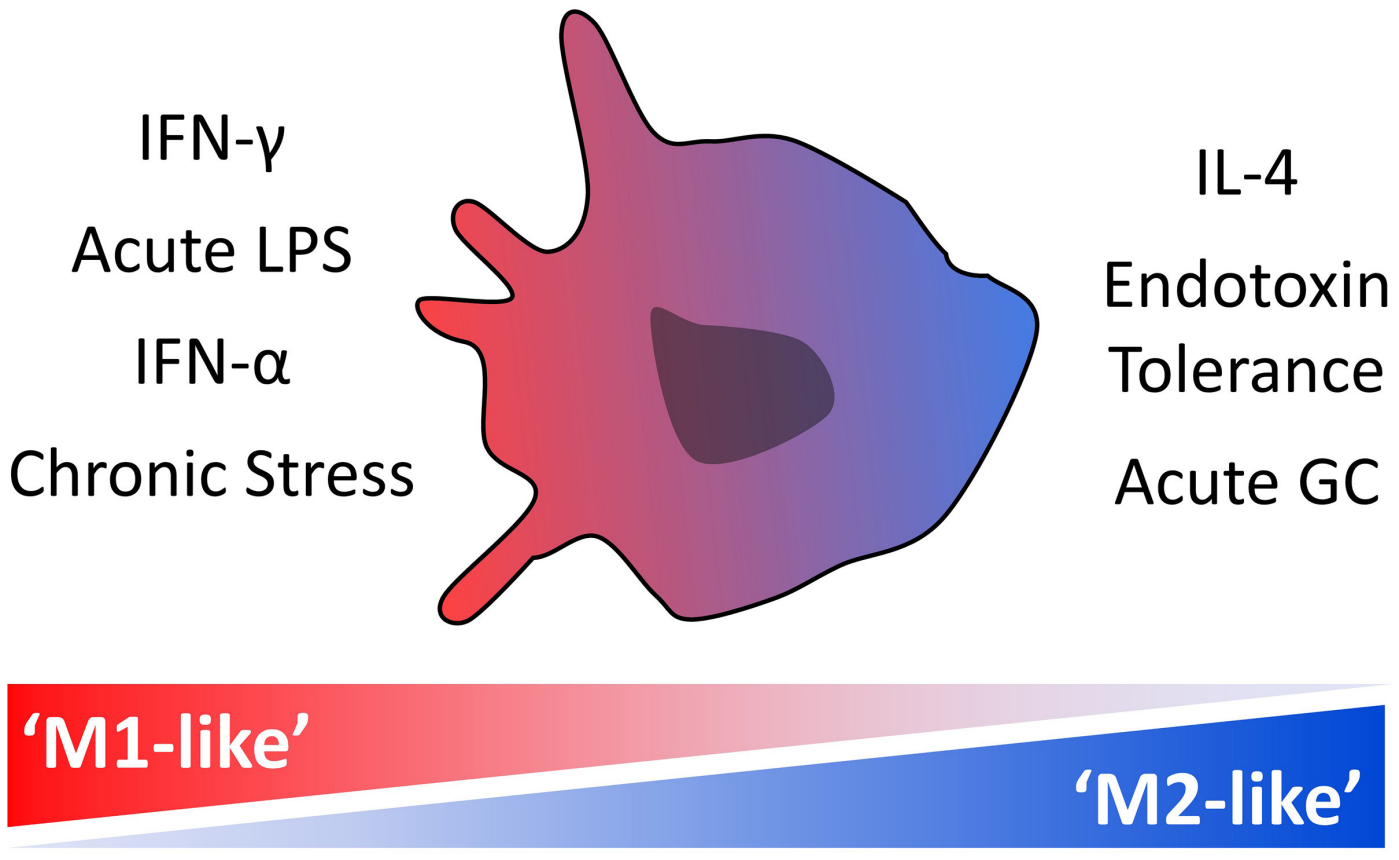
Stimuli and physiological states associated with monocyte “M1-like” and “M2-like” phenotypes. Monocytes exist along a spectrum of activation states that are dependent on the overall combination of environmental signals, with specific transcriptional signatures indicative of a more proinflammatory “M1-like” phenotype and others of a more anti-inflammatory or protective “M2-like” phenotype.

The functional phenotype of myeloid cells, including monocytes and macrophages, is highly sensitive to microenvironmental cues [Figure 1; (17, 18)]. The induction of a proinflammatory phenotype, frequently termed “M1” or “M1-like,” is transduced by activation of the JAK-STAT1 pathway by interferon-gamma (IFN-γ) alone or in conjunction with an NF-κB activating stimulus such as lipopolysaccharide (LPS) (19). Other stimuli or physiological states that are associated with an “M1-like” monocyte phenotype are type I interferons (IFN-α/β) and chronic stress (20, 21). “M2-like” monocytes and macrophages, which are skewed toward an anti-inflammatory/tissue remodeling phenotype, are associated with various physiological states and stimuli including the cytokines interleukin (IL)-4 and IL-13, acute stress/glucocorticoids, and endotoxin tolerance, which is induced by high levels of NF-κB activating stimuli such as LPS in the absence of IFN-γ (22). While these terms “M1-like” and “M2-like” imply bipolarity, a spectrum or multipolar model of possible activation states and functions that are dependent on the overall combination of environmental signals is now considered to be more conceptually useful (17). The extent to which opposing phenotypes can co-occur *in-vivo* is unclear, and individual stimuli can have both synergistic and antagonistic effects. For example, the “M1-like” stimulus, IFN-γ, induces epigenetic changes in myeloid cells that affect their subsequent responses to stimuli, inducing open chromatin at proinflammatory gene promoters that leads to an increased response to proinflammatory stimuli, but silencing promoters of genes induced by the “M2-like” stimulus IL-4 and reversing endotoxin tolerance (19, 23, 24). Transcriptomics have been increasingly used to characterize myeloid cell phenotypes in response to a variety of environmental signals (25, 26). The rich datasets generated under homeostatic conditions as well as in the context of injury and illness reveal a previously underappreciated degree of complexity in the myeloid cell transcriptional landscape, functional capacity and role in pathophysiological processes.

In clinical studies of schizophrenia, gene expression profiling of circulating cells has predominantly been carried out using heterogeneous cell populations. Some results demonstrate increased expression of proinflammatory cytokines such as IL-6 and (tumor necrosis factor alpha) TNF-α, and show activation of the NF-κB pathway in schizophrenia (27, 28), whereas other studies find no differences in expression of these immune markers (29). Further complicating this picture and likely contributing to conflicting results, independent studies and meta-analyses indicate that changes to the immune system do not remain static over the course of the illness. For example, we previously showed that expression of an IFN-γ signature was decreased in circulating blood cells earlier in illness and positively correlated with duration of schizophrenia (30). Similarly, a combined innate immunity score was decreased in those with recent onset schizophrenia, but increased in chronic schizophrenia (31), and one recent meta-analysis found minimal differences in immune markers in first-episode psychosis, whereas illness progression was associated with immune alterations (32). However, there are conflicting data as other meta-analyses have found immune alterations, including elevated proinflammatory cytokines, in first-episode, acute and chronic schizophrenia (1, 33, 34). Overall, these results support a possible shift in immune dysregulation over the course of illness, potentially contributing to the high variability seen in the cytokine literature in schizophrenia. Notably, these studies were also carried out using heterogeneous immune cell populations or serum and thus the contribution of specific cell types is unknown, also further complicating interpretation of findings related to immune measures.

Here, focusing on chronically symptomatic participants with schizophrenia, we carried out RNA sequencing of isolated monocytes, with the aim of clarifying the pattern of immune dysregulation in these critical orchestrators of an inflammatory response. Utilizing a transcriptomic approach allowed for the detection of perturbations in the overall pattern of gene expression and isolation of relevant signaling pathways, not afforded by an individual marker approach. Our primary analysis compares participants with schizophrenia and non-psychiatric controls. Additionally, because our previous findings could indicate a shift in monocyte phenotype in relation to illness duration, an additional exploratory analysis was carried out in which participants with schizophrenia were split into subgroups based on illness duration. In our previous analysis shifting immune signatures were associated with both illness acuity and duration (30). In this small sample we focus on individuals with chronic and persistent schizophrenia, but no acute exacerbation of symptoms, controlling for illness acuity as a variable. Gene set enrichment analysis (GSEA) was used to test for enrichment of preselected phenotypic signatures in isolated monocytes from participants. We focused on seven previously defined monocyte-macrophage transcriptional signatures, associated with “M1-like” or “M2-like” activation states (see **Table 2**). Finally, transcription factor enrichment analyses (TFEA) were carried out to determine which signaling systems may be driving differences in monocyte gene expression.

## Materials and Methods

### Participant Characteristics

Participant demographic and clinical characteristics are outlined in Table 1. Participants with schizophrenia who met Diagnostic and Statistical Manual of Mental Disorders (DSM)-IVtr diagnostic criteria (41) were recruited from a large Urban University Medical Center that included referrals from community treatment facilities. All participants with schizophrenia took part as outpatients. Control participants were recruited from the surrounding urban community and had no current or past history of psychiatric illness. Simple random sample strategy was applied. The study was approved by the Institutional Review Board of the University of Illinois at Chicago (IRB2012-0113) and was conducted in accordance with the latest version of the Declaration of Helsinki (42). All participants gave signed written consent prior to initiation of any research procedures. Inclusion criteria consisted of individuals between the ages of 21–60 years who met DSM-IVtr diagnostic criteria for schizophrenia as well as control subjects with no current or previous history of a major psychiatric disorder. Exclusion criteria included current fever or known infection, treatment with VPA, carbamazepine, or clozapine in the previous 30 days, substance dependence, current pregnancy, seizure disorders, immune system disorder, or neurological conditions. While no participants reported a history of immune system problems, one participant in each group reported a history of rheumatic illness, though no lab or clinical evaluation was conducted. Consensus diagnoses were determined by both the clinical and research team using the Structured Clinical Interview for DSM-IVTR (41) and available collateral information. All participants with schizophrenia were currently experiencing chronic and persistent symptoms, but were without an acute exacerbation of psychosis. Clinical symptomology was measured using the positive and negative syndrome scale (PANSS) (43). The PANSS was administered to participants by trained licensed clinical research faulty. Antipsychotic use for participants with schizophrenia was converted to chlorpromazine equivalents (CPZE) (44, 45). For the exploratory analyses to assess the effects of illness duration on monocyte gene expression, the sample of participants with chronic schizophrenia was arbitrarily split into two illness duration subgroups; “medium illness duration” and “long illness duration” (Table 1). Illness duration in the medium illness duration group ranged from 25 to 31 years and in the long illness duration group from 32 to 46 years. Group differences between all participants with schizophrenia and controls were assessed using independent samples *t*-tests and chi-square tests. Group differences between two illness duration groups and controls were assessed using one-way ANOVA followed by Tukey's *post-hoc* tests if significant. Differences in illness duration between the two illness duration groups were confirmed using an independent samples *t*-test.

**Table 1:** Participant demographics and clinical characteristics for primary and subgroup analyses.

		**Control**	**Schizophrenia**	**Illness duration subgroup**	
				**Medium**	**Long**
Total (n)		14	14	7	7
Age (M ± SD)		49.00 ± 11.64	51.79 ± 6.34	51.29 ± 7.18	52.29 ± 5.91
Sex	Female (n)	7	7	4	3
	Male (n)	7	7	3	4
Race	Caucasian, non-hispanic (n)	2	5	2	3
	Black, non-hispanic (n)	12	8	4	4
	Hispanic (n)	0	1	1	0
BMI (M ± SD)		28.91 ± 8.07	32.91 ± 12.67	33.70 ± 5.38	32.11 ± 4.53
Illness duration (M ± SD)		N/A	32.36 ± 6.86	27.29 ± 2.14^**^	37.43 ± 6.11^**^
Age of symptom onset (M ± SD)		N/A	19.43 ± 7.32	24.00 ± 6.71^*^	14.86 ± 5.58^*^
PANSS	Positive (M ± SD)	8.93 ± 1.69	29.71 ± 3.58^#^	28.71 ± 3.45^#^	30.71 ± 3.68^#^
	Negative (M ± SD)	9.07 ±1.86	22.00 ± 5.56^#^	21.71 ± 5.53^#^	22.29 ± 6.02^#^
	General (M ± SD)	24.07 ± 3.02	49.00 ± 7.63^#^	48.43 ± 6.58^#^	49.57 ± 9.05^#^
Current antipsychotic treatment (oral) (n)^‡^		0	13 (10)	6 (3)	7 (7)
CPZE (M ± SD)		N/A	599.17 ± 520.84	336.11 ± 235.75	711.91 ± 582.20
Nicotine use (n)		6	5	3	2
Immune-modulatory medications statins, metformin (n, n)		2,0	4,1	2,1	2,0

# *p < 0.001 comparison with controls*.

* *p < 0.05*,

** *p < 0.01 comparison of medium and long illness duration groups*.

*M, mean; SD, standard deviation*.

‡ *Medium illness duration: Haloperidol LAI (1), Paliperidone LAI (1), Aripriprazole LAI (1), Olanzapine (1), Risperidone (1), Quetiapine (1); Long illness duration: Fluphenazine (1), Haloperidol (1), Risperidone (1), Olanzapine (2), Lurasidone (1), Ziprasidone (1)*.

### Sample Collection and RNA Extraction

The blood draw and peripheral blood mononuclear cell (PBMC) isolation for each participant were carried out according to previously described protocols (46). The blood draw was conducted between the hours of 8 and 9 A.M. for all participants, following overnight fasting. Initiation of PBMC and monocyte isolation protocols took place shortly after blood sample collection. Cluster of differentiation 14 (CD14), a cell surface marker expressed on monocytes and macrophages (47), was used to isolate monocytes from the PBMC cell suspension. Cells were labeled with anti-CD14 coated microbeads, and positively selected using magnetic cell sorting according to the manufacturers protocol (Miltenyi Biotech). Monocytes were washed with phosphate buffered saline, pelleted by centrifugation, and stored at −80°C prior to RNA extraction. RNA was extracted from the CD14+ primary monocytes using the Qiagen miRNeasy mini kit with Qiagen on-column DNase treatment. RNA integrity was assessed using the Agilent tapestation, and was considered satisfactory for 3' library preparation.

### Library Preparation and Sequencing

Library preparation and sequencing were carried out at the University of Illinois at Chicago Core Facilities. The cDNA library was prepared by the Core Genomics Facility using the Lexogen QuantSeq FWD kit and cDNA libraries were sequenced using an Illumina HiSeq system by DNA Services. Quant-seq, also known as 3'Tag-Seq, is a protocol to generate low-noise gene expression profiling data. In contrast to traditional RNA-Seq, which generates sequencing libraries from the whole transcripts, 3'Tag-Seq only generates a single initial library molecule per transcript, complementary to 3′-end sequences. This technology reduces the number of sequencing reads needed for accurate quantification and negates the need for the gene length normalization steps that alternative RNA-Seq methodologies require.

### Differential Gene Expression

Read count matrices were generated using the Bluebee bioinformatics pipeline. Briefly, “bbduk” was used for adapter trimming, “FastQC” for read quality control, “STAR” for alignment and “HTSeq” for read counting. Genes with read counts with a mean expression under 10 across all samples were removed, as were genes expressed in < 75% of the participant sample. Differential gene expression (DGE) analyses were conducted in Bluebee using “DESeq2” (Bioconductor) (48).

### Gene Set Enrichment Analysis

For each comparison, a GSEA rank file was first created by ranking genes from the greatest increase to the greatest decrease in expression using the DESeq2 Wald statistic pair-wise comparison output (49). Seven gene sets were compiled, a transcriptional signature representative of each of the previously described cellular phenotypes (Table 2). For clarity, specific “signatures” are denoted with quotation marks, and the associated term *gene set* italicized. The analysis was carried out using the Broad Institute GSEA Preranked software with the recommended default settings of 1,000 permutations and weighted enrichment. For each preselected gene set, an enrichment score (ES) was computed. The ES is calculated by walking down the rank file and creating a running score such that when a gene from the gene set being analyzed is in the rank file the score increases, and if not present the score decreases. The resulting ES, the maximum deviation from zero, indicates whether or not a gene set is enriched amongst the genes at either end of the rank file. The ES is first calculated for each gene set individually, followed by normalization to allow for comparison between gene sets of different sizes. A *gene set* was considered significantly enriched if both the nominal *p*-value was under 0.05 and the False Discovery Rate (FDR) under 0.25. “Leading-edge” analyses were carried out for significantly enriched genes sets in each comparison, again using the Broad Institute GSEA Software (49). The *leading-edge subset of genes* (italicized for clarity) for a differentially enriched *gene set* consists of the genes leading up to, and therefore contributing to, the maximum ES. Any *leading-edge subset genes* that are present in two or more of the significantly enriched *gene set*s are highlighted. Two additional GSEA were conducted. First, GSEA was conducted using the Molecular Signatures Database (MSigDB) “hallmark” *gene sets* list (50) in place of the 7 predefined *gene sets* listed in Table 2. This collection of 50 low redundancy and robust gene sets was compiled in 2015 using gene lists available in the Molecular Signatures Database. Second, GSEA was conducted using illness duration as a continuous variable. Here, the GSEA rank file is the correlation of gene expression with illness duration using Pearson's ranking metric. Note that this analysis uses only data from participants with schizophrenia, excluding control participants.

**Table 2:** *Gene set*s, i.e., transcriptional signatures, representative of specific “M1-like” and “M2-like” monocyte phenotypes compiled from the published literature that were used for GSEA.

	**Gene set**	**No. of Genes**	**Cell type**	**Conditions**	**Data acquisition**	**References**
M1-like	IFN-γ signature	500	Human primary monocyte-macrophages	Culture IFN-γ 24 h	RNA-seq	(35)
	LPS acute signature	330	Human primary monocyte-macrophages	Culture LPS 4 h	RNA-seq	(36)
	IFN-α signature	200	Human primary monocyte-macrophages	Culture IFN-α 4 h	Microarray	(37)
	Chronic stress signature	315	Human primary monocytes	*In-vivo* chronic caregiver stress	Microarray	(20)
M2-like	IL-4 Signature	200	Human primary monocyte-macrophages	Culture IL-4 24 h	Microarray	(38)
	ET Signature	76	Human PBMCs	*In-vivo* Sepsis	Microarray	(39)
	GC Acute Signature	87	Human primary monocyte-macrophages	Culture dexamethasone 1–24 h	Microarray	(40)

*ET, endotoxin tolerance; GC, glucocorticoid*.

### Transcription Factor Enrichment Analysis

Transcription factor enrichment analysis was carried out for each of the IFN-γ signature *leading-edge subset genes*. The chromatin immunoprecipitation (ChIP)-X database (ChEA 2016) was used and TFEA implemented in Enrichr to carry out over representation analysis (51–53). The ChIP-X database contains lists of transcription factor target genes compiled using results of published genome-wide transcription factor ChIP experiments (645 gene lists at the time of analysis). This method determines whether genes that are regulated by a particular transcription factor are over-represented within the *leading-edge subset genes*. The data are presented using the combined score output, which is representative of the *p*-value and the z-score that assesses deviation from the expected rank. Because this database contains results from multiple organisms and cell lines the same transcription factor may be repeated in the enrichment results. Additionally, a further complementary analysis was conducted in which GSEA was carried out using the Broad Institute software for the medium vs. high ranked gene list using the entire ChIP-X database as the *gene set*s list.

## Results

### Demographics

#### Participants With Schizophrenia Compared to Controls

There was no difference in age (*t*_26_ = −0.79, *p* = 0.44), sex [χ^2^(1) = 0.00, *p* = 1.00], race [χ^2^(2) = 3.09, *p* = 0.21] or body mass index (BMI) (*t*_26_ = −1.00, *p* = 0.33) when comparing participants with schizophrenia and controls. PANSS positive (*t*_26_ = −19.64, *p* < 0.001), negative (*t*_26_ = −8.25, *p* < 0.001) and general (*t*_26_ = −11.37, *p* < 0.001) subscale scores were greater in participants with schizophrenia than non-psychiatric controls.

#### Illness Duration Subgroup Analysis

The control participants and participants with schizophrenia in the medium and long illness duration groups demonstrated no differences in age [*F*_(2, 25)_ = 0.32, *p* = 0.73], sex [χ^2^(2) = 0.29, *p* = 0.87], race [χ^2^(4) = 5.37, *p* = 0.25], or BMI [*F*_(2, 25)_ = 0.52, *p* = 0.60]. Again, PANSS positive [*F*_(2, 25)_ = 200.12, *p* < 0.001], negative [*F*_(2, 25)_ = 32.84, *p* < 0.001] and general [*F*_(2, 25)_ = 62.54, *p* < 0.001] scores were greater in both illness duration groups relative to controls (*p* < 0.001 for all subscales), but there were no differences in PANSS scores between the two illness duration groups. As intended, illness duration was significantly greater in the long illness duration group than the medium illness duration group (*t*_12_ = −4.15, *p* = 0.004). Additionally, age of onset was lower in the long illness duration subgroup (*t*_12_ = −2.77, *p* = 0.017). There was no difference in CPZE between the two illness duration groups for participants treated with oral antipsychotics including or excluding the untreated participant (*t*_9_ = −1.47, *p* = 0.17; *t*_8_ = −1.05, *p* = 0.32, respectively).

### Transcriptome Analysis in Participants With Schizophrenia Compared to Controls

**Figure 2:**
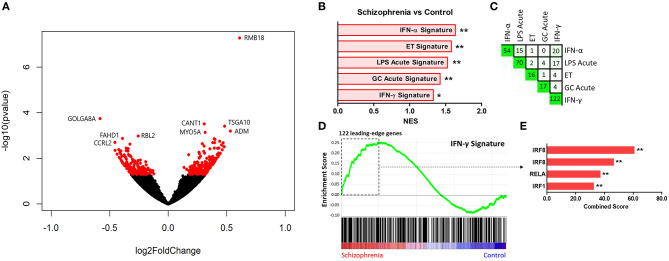
**(A)** Volcano plot of DGE: schizophrenia vs. controls. On the x-axis is log fold change of genes in participants with schizophrenia compared to controls, points to the right of 0 represent genes that are increased, and points to the left of 0 genes that are decreased, in schizophrenia compared to controls. Statistical significance is displayed on the y-axis, and *p* < 0.05 are labeled in red. Select genes with both high fold change and significance are labeled. **(B)** Signatures that were enriched in the schizophrenia group compared to controls. **FDR < 0.05, *FDR < 0.25. NES, normalized enrichment score. **(C)** Leading-edge analysis for signatures enriched amongst genes overexpressed in schizophrenia. The numbers indicate the number of *leading-edge subset genes*, for example there were 54 *leading-edge subset genes* for the “IFN-α signature,” as well as the number of overlapping *leading-edge subset genes* between the corresponding signatures, for example 15 genes overlap in the “IFN-α signature” and the “LPS acute signature” *leading-edge subset genes*. This number is also represented by the green gradient. **(D)** Enrichment plot for the IFN-γ signature in participants with schizophrenia compared to controls. Genes are ranked from greatest increase to the greatest decrease in expression in schizophrenia compared to controls. Black lines indicate the location of genes that are in both the ranked list and *gene set*. **(E)** Top four results from the TFEA of the *leading-edge subset genes* in the IFN-γ signature. **p < 0.001 and adjusted *p* < 0.001.

Comparison of participants with schizophrenia and controls by DESeq2 revealed 389 protein-coding genes differentially expressed in primary monocytes, 214 increased and 175 decreased in participants with schizophrenia at *p* < 0.05 (Figure 2A). A rank file was generated from the DGE output and GSEA carried out to determine whether any of the seven selected gene sets were enriched in schizophrenia compared to control participants and vice versa. The IFN-γ, IFN-α, ET, LPS acute, and GC acute signatures were enriched amongst the genes increased in schizophrenia (Figures 2B,D). These results indicate that both the type I (“IFN-α signature”) and type II (“IFN-γ signature”) IFN signatures, the primary drivers of JAK-STAT1 signaling, are increased in all participants with schizophrenia compared to controls. Interestingly, genes that are characteristic of an *in-vivo* endotoxin tolerant state (“ET signature”) and genes that are expressed following *in-vitro* acute LPS challenge (“acute LPS signature”) were also both enriched in schizophrenia. Finally, there was also enrichment of the cellular response to glucocorticoids (“GC acute signature”), but no evidence of enrichment of the *in-vivo* transcriptional changes that are observed in individuals experiencing chronic stress (“chronic stress signature”). Leading-edge analysis for the enriched signatures is shown in Figure 2C. TFEA for the 122 IFN-γ signature leading-edge subset genes demonstrated that interferon regulated factor 8 (IRF8) is the most likely transcription factor candidate driving the enrichment of this signature in schizophrenia (Figure 2E). Also enriched were RELA, the NF-κB p65 subunit, and IRF1, which acts alongside IRF8 to mediate many of the genomic effects downstream of IFN-γ induced JAK-STAT1 signaling. An additional GSEA carried out using the MSigDB “hallmark” gene set list in place of the 7 predefined gene sets demonstrated strongest enrichment of “hallmark_interferon_alpha_response,” “hallmark_interferon_gamma_response,” and “hallmark_oxidative_phosphorylation” (Supplementary Table 1).

### Exploratory Transcriptome Analysis in Subgroups Based on Illness Duration

**Figure 3:**
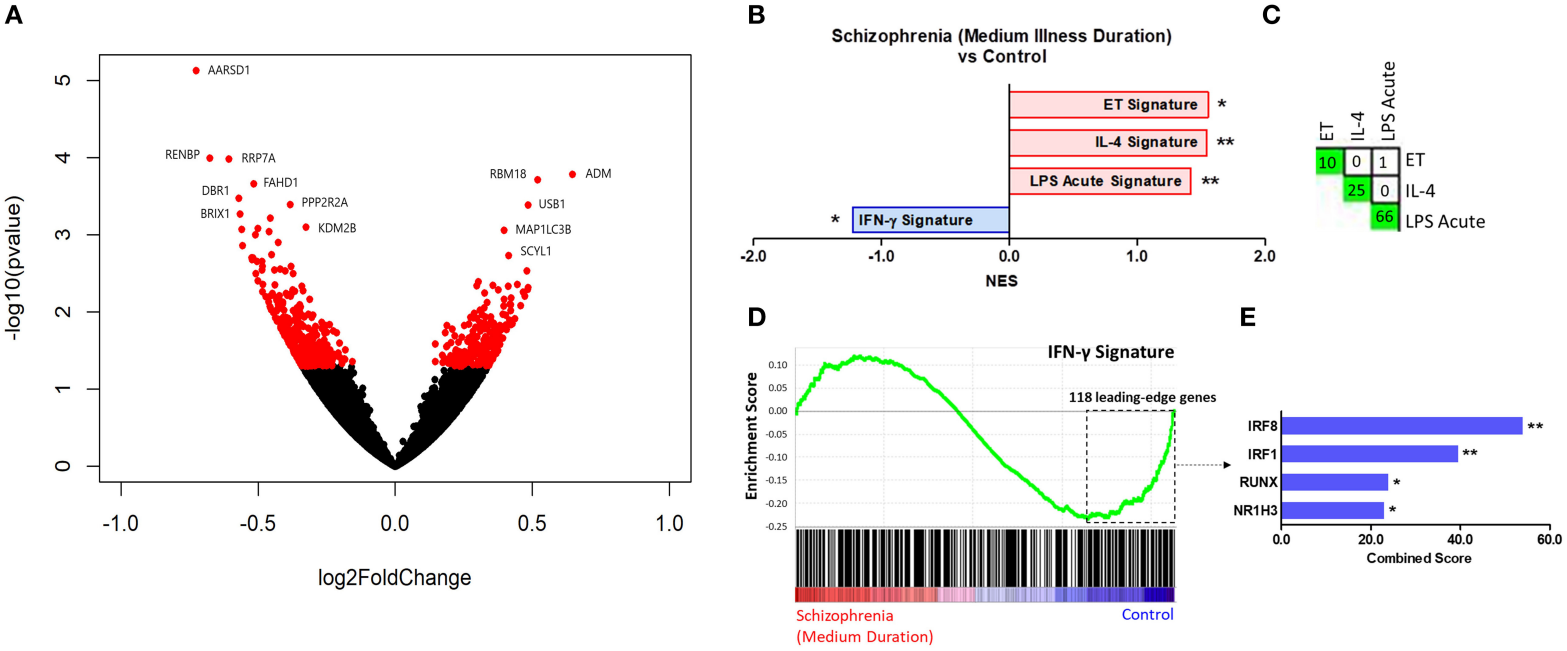
**(A)** Volcano plot of DGE: medium illness duration vs. controls. *p* < 0.05 labeled red. **(B)** Signatures that were enriched in the medium illness duration group compared to controls. **FDR < 0.05, *FDR < 0.25. **(C)** Leading-edge analysis for signatures enriched amongst genes increased in the medium illness duration group compared to controls. The number and green gradient indicate overlapping *leading-edge subset genes* between the corresponding signatures. **(D)** Enrichment plot for the IFN-γ signature in the medium illness duration group compared to controls. Genes are ranked from greatest increase to the greatest decrease in expression in the medium illness duration group compared to controls. Black lines indicate the location of genes that are in both the ranked list and gene set. **(E)** Top four results from the TFEA of the *leading-edge subset genes* in the IFN-γ signature. **p < 0.001 and adjusted *p* < 0.001, *adjusted *p* < 0.01.

**Figure 4:**
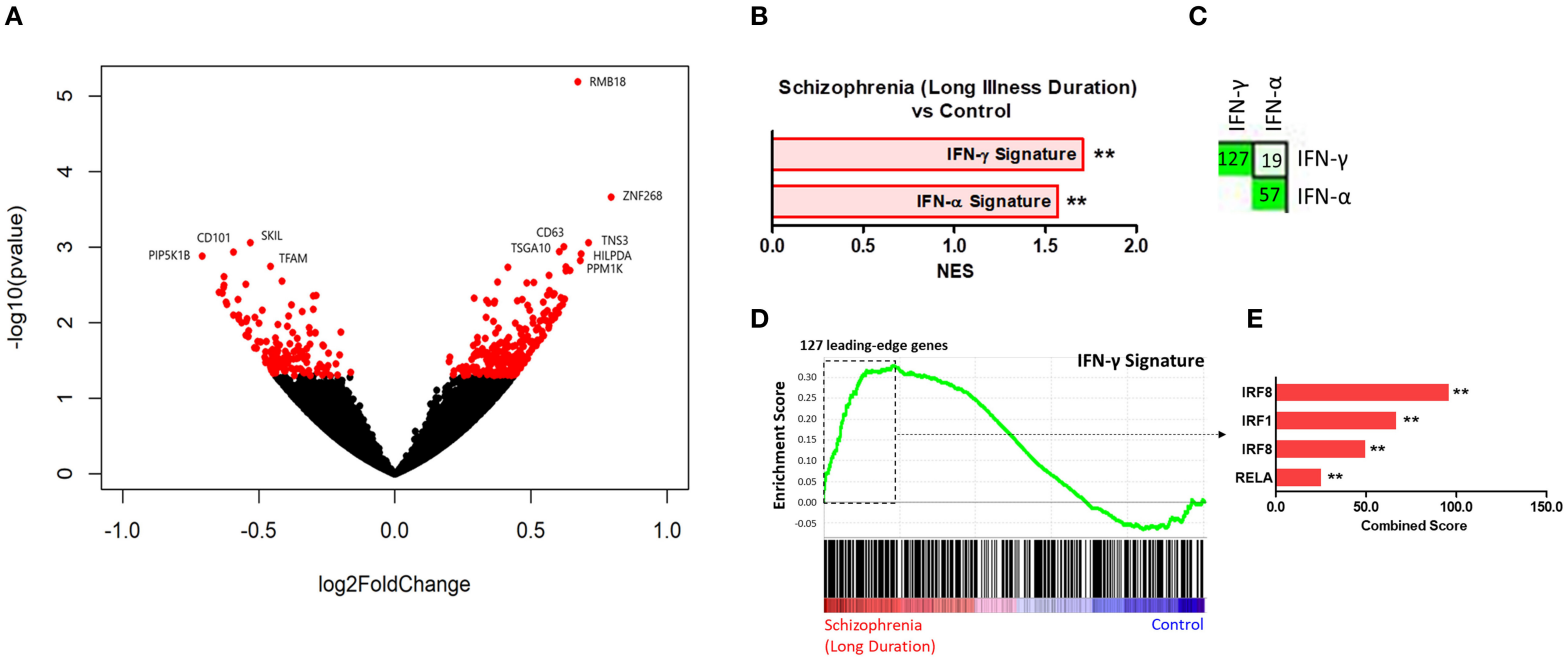
**(A)** Volcano plot of DGE: long illness duration participants vs. controls. *p* < 0.05 labeled red. **(B)** Signatures that were enriched in the long illness duration group compared to controls. **FDR < 0.05, *FDR < 0.25. **(C)** Leading-edge analysis for signatures enriched amongst genes increased in the long illness duration group compared to controls. The number and green gradient indicate overlapping *leading-edge subset genes* between the corresponding signatures. **(D)** Enrichment plot for the IFN-γ signature in the long illness duration group compared to controls. Genes are ranked from greatest increase to the greatest decrease in expression in the long illness duration group compared to controls. Black lines indicate the location of genes that are in both the ranked list and gene set. **(E)** Top four results from the TFEA of the *leading-edge subset genes* in the IFN-γ signature. **p < 0.001 and adjusted *p* < 0.001.

**Figure 5:**
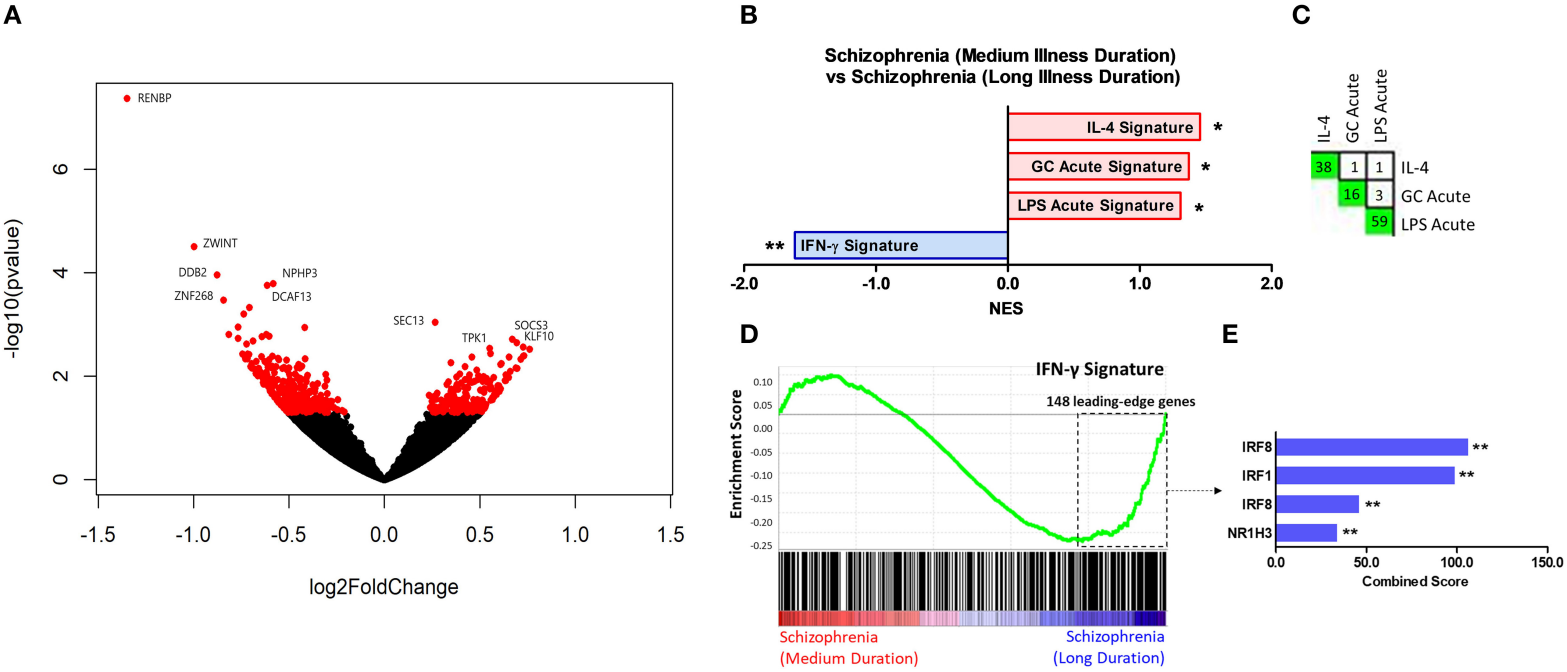
**(A)** Volcano plot of DGE: medium vs. long illness duration participants. *p* < 0.05 labeled red. **(B)** Signatures that were enriched in the medium illness duration group compared to the long illness duration group. **FDR < 0.05, *FDR < 0.25. **(C)** Leading-edge analysis for signatures enriched amongst genes increased in the medium illness duration group compared to controls. The number and green gradient indicate overlapping *leading-edge subset genes* between the corresponding signatures. **(D)** Enrichment plot for the IFN-γ signature in the long illness duration group compared to controls. Genes are ranked from greatest increase to the greatest decrease in expression in the long illness duration group compared to controls. Black lines indicate the location of genes that are in both the ranked list and *gene set*. **(E)** Top three results from the TFEA of the *leading-edge subset genes* in the IFN-γ signature. **p < 0.001 and adjusted *p* < 0.001.

**Figure 6:**
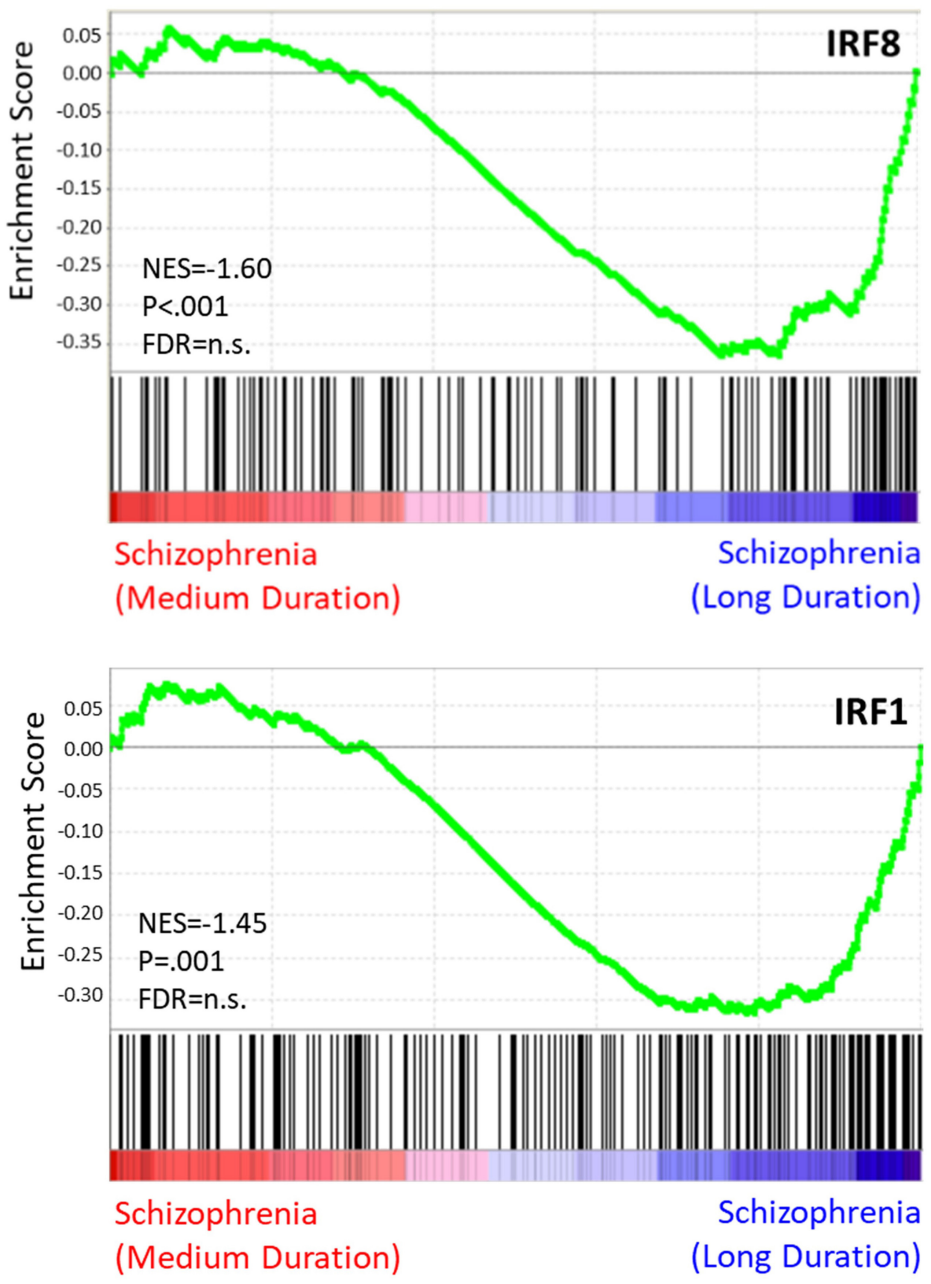
TF enrichment plots: medium vs. long illness duration. Genes are ranked from greatest increase to the greatest decreased in expression in the long illness duration group compared to controls. Black lines indicate the location of genes that are in both the ranked list and *gene set*.

Results of the exploratory illness duration subgroup analyses are presented in Figures 3–**5**. GSEA revealed that the IFN-γ signature was enriched amongst the genes increased in controls compared to participants with schizophrenia in the medium illness duration group (Figures 3B,D). In contrast, the ET, IL-4, and LPS acute signatures were all enriched amongst the genes increased in the medium illness duration group of participants with schizophrenia compared to controls. There was no enrichment of either the GC acute or chronic stress signatures. In participants in the long illness duration group GSEA demonstrated only enrichment of the IFN-γ and IFN-α signatures compared to controls (Figures 4B,D). Finally, the IFN-γ signature was enriched in the group of participants with a long compared to medium illness duration, whereas the IL-4, GC acute and LPS acute signatures were enriched in the participants with a medium compared to long illness duration (Figures 5B,D). For each TFEA of the *leading-edge subset genes* driving enrichment of the IFN-γ signature IRF8 and IRF1 were the most strongly enriched (Figures 3–5 part E). RUNX, a transcription factor involved in survival and differentiation of monocyte/macrophages in addition to cell adhesion (54), NR1H3, a liver X receptor known to negatively regulate inflammation (55), and RELA were also enriched in some of the comparisons. Next, GSEA was carried out using the entire ranked gene list for the medium vs. high illness duration subgroup comparison and the 2016 ChIP-X database as the *gene set* file in order to determine which transcription factors may be driving the decreased expression in the medium illness duration group, i.e., without constraining the input to IFN-γ *leading-edge subset genes*. The results revealed that IRF8 and IRF1 regulated genes were once again the top two enriched sets (Figure 6), though no set was significant at an FDR < 0.25. This strengthens the implication that these signaling components are involved in the monocyte phenotype shifts that occur with increasing illness duration in schizophrenia. Finally, results of the GSEA with MSigDB “hallmark” *gene sets* list demonstrated strongest enrichment of “hallmark_oxidative_phosphorylation” in the medium illness duration subgroup compared to controls, the “hallmark_interferon_alpha_response” and “hallmark_interferon_gamma_response” in the long illness duration subgroup compared to controls and “hallmark_tnfa_signaling_via_nfkb” in the medium compared to long illness duration subgroups (Supplementary Table 1). Finally, to include non-psychiatric controls in the exploratory analysis required treatment of illness duration as a categorical variable, splitting participants into two illness duration groups. To better understand the pattern of signature enrichment in relation to illness duration as a continuous variable GSEA was carried out in the participants with schizophrenia only focused on the correlation of gene expression with illness duration, which highlighted enrichment of the “IFN- γ signature” and “IFN-α signature” amongst the genes with the strongest positive correlation with illness duration (Supplementary Table 2).

## Discussion

As research highlights the role of peripheral immune activity in CNS homeostasis, neuroinflammation, and neuropsychiatric disorders, an understanding of the changes to immune cell phenotype and function in psychosis becomes increasingly important (2, 56). To our knowledge, this is the first investigation of the monocyte transcriptome in schizophrenia using genome-wide RNA sequencing. We demonstrate enrichment of both “M1-like” and “M2-like” monocyte phenotype signatures in chronic schizophrenia relative to controls, and the results of the exploratory analysis indicate a shift in monocyte phenotype in later stages of illness.

Our primary analyses compared participants with chronic schizophrenia to non-psychiatric controls. Using predefined gene sets representative of both proinflammatory “M1-like” and protective or tissue remodeling “M2-like” functional signatures in myeloid cells (Figure 1, Table 1), we show that monocytes from the participants with schizophrenia have concurrent increased enrichment of both “M1-like” and “M2-like” transcriptional signatures relative to controls. The primary gene sets representative of an “M1-like” phenotype were the “IFN-γ signature” and “LPS acute signature.” IFN-γ, the type II interferon, is a well-characterized and vital myeloid cell “M1-like” polarizing stimulus, known to act both synergistically and antagonistically with other stimuli present in the cells' environment (19). TLR4 mediated NF-κB signaling, which can be activated by acute LPS treatment, is a key proinflammatory pathway that controls expression of cytokines and other inflammatory mediators and acts synergistically alongside IFN-γ mediated JAK-STAT1 signaling to contribute to an proinflammatory “M1-like” phenotype (19). Both signatures were enriched, though the “IFN-γ signature” satisfied only the less strict FDR threshold. The type I interferon, IFN-α (“IFN-α signature”), was also included in the analysis due to substantial overlap with the “IFN-γ signature” and interestingly, was the most strongly enriched signature in the participants with schizophrenia.

IL-4 is an important cytokine that induces a canonical “M2-like” myeloid cellular phenotype, skewed toward anti-inflammatory and tissue remodeling functions (19). This phenotype is characterized in part by decreased JAK-STAT1 signature gene expression, and IFN-γ and IL-4 are known have antagonistic actions in monocyte/macrophages (57). The “endotoxin tolerance (ET) signature,” another of the “M2-like” signatures examined, is induced in response to high levels of NF-κB activating stimuli (39, 58). Though there were no differences in enrichment of the “IL-4 signature,” the “ET signature” was enriched in schizophrenia relative to non-psychiatric controls. Finally, stress has been demonstrated to impact the phenotype of circulating monocytes and is a risk factor for psychosis as well as important consideration in adults with major psychiatric illness. While acute stress is predominantly associated with an anti-inflammatory “M2-like” transcriptional response, chronic stress is associated with a specific “genomic fingerprint” in monocytes, with increased “M1-like” pro-inflammatory activity and glucocorticoid resistance (20). The “GC acute” signature was enriched overall in the schizophrenia group whereas the “chronic stress” signature did not demonstrate enrichment in any of the comparisons. Thus, the inflammatory profile and glucocorticoid resistance that can develop as a result of chronic stress does not map onto the changes seen in immune cell signaling in schizophrenia.

Furthermore, using a more unbiased approach in which all 50 of the hallmark gene sets from the molecular signatures database were assessed using GSEA, we found strongest enrichment of both the “IFN-α response” and “IFN-γ response” signatures in schizophrenia relative to controls. Of other notable signatures relevant to immune signaling, the “inflammatory response” satisfied a less stringent FDR < 0.25 but not <0.05, and the “IL-6 JAK-STAT3 signaling” and “TNF-α signaling via NF-κB” signatures were not enriched in schizophrenia.

Because our previous findings and several other studies indicate that alterations to circulating immune markers change throughout the course of illness in psychosis (30–32), we carried out additional exploratory analyses using illness duration to separate participants with schizophrenia into medium and long illness duration subgroups. The “IFN-γ signature” showed decreased enrichment in the medium illness duration group compared to controls and to the long illness duration group, whereas the signature was increased in the long illness duration group compared to controls. These findings, using an independent cohort and a single immune cell type, are consistent with our previous data demonstrating a shift in the circulating leukocyte IFN-γ signature over the course of illness in psychosis, and indicate that monocytes drive or contribute to this phenomenon.

In the long illness duration group this increase was also mirrored by enrichment of the type I IFN (“IFN-α signature”) signature, whereas the decreased IFN signature in the medium illness duration group was specific to IFN-γ. Enrichment of the “LPS acute signature” was seen in the medium, but not the high, illness duration group. Thus, while both the IFN-γ and LPS acute signatures were elevated overall in schizophrenia, contrary to our expectations they diverge in relation to illness duration, with enrichment of the LPS acute signature present only earlier in illness and enrichment of the IFN-γ signature present only later in illness. Examination of the endotoxin tolerance “ET signature” revealed an enrichment in the medium illness duration group, indeed opposing the IFN-γ signature, but somewhat counterintuitively mirroring the “LPS acute signature,” with minimal overlap of genes contributing to the enrichments. The IL-4 signature was enriched in the medium illness duration group, the predicted pattern of enrichment opposing that seen for the IFN-γ signature.

Thus, in the participants who had a medium illness duration this suppressed “M1-like” IFN-γ signature was accompanied predominantly by “M2-like” anti-inflammatory and tissue remodeling (ET, IL-4, GC) signatures, yet also demonstrated an enrichment of the “M1-like” (LPS acute) signature. While IFN-γ and LPS are both described in the literature as proinflammatory stimuli, acting synergistically to induce an “M1-like” myeloid cell phenotype, *in-vivo* these two signatures did not show coordinated expression with one another in circulating monocytes. In the long illness duration subgroup the monocyte phenotype consisted of an interferon response (both type I and II) signature enrichment only. The results of the hallmark gene sets analysis in the illness duration subgroups mirrored some of these findings using the predefined gene sets with strongest enrichment of the interferon alpha and gamma responses in the long illness duration subgroup and strongest enrichment of TNF-α signaling via NF-κB (in lieu of the LPS acute response) when comparing the medium to long illness duration subgroups. An additional analysis including illness duration as a continuous variable, conducted in the participants with schizophrenia only, demonstrated enrichment of the “IFN-γ signature” and “IFN-α signature” amongst the gene with the strongest positive correlation with illness duration supporting the results of the categorical subgroup analysis. These results indicate a shift in pro- and anti-inflammatory monocyte phenotypic signatures with illness progression, and highlight the importance of considering factors such as illness duration when investigating immune dysfunction in major psychiatric illness.

These data further demonstrate another important point, which is that *in-vivo*, even an isolated cell population can display a mixed cellular phenotype with both pro- and anti-inflammatory functional signatures. A concurrent enrichment of proinflammatory and endotoxin tolerance signatures was also seen in circulating immune cells from participants with sepsis (39). The authors hypothesized that the presence of both signatures could be due to the mixed cell population from which they derived the signature. As here we describe the same phenomenon in an isolated cell type, an related explanation is that a mixed pro-and anti-inflammatory signature enrichment may be due to the fact that circulating monocytes are at different developmental timepoints and thus enrichment is representative of different stages of response to the same microenvironmental signals, or, alternatively, that the responses represent heterogeneous monocyte responses (59). Importantly, it is also possible that individual cells may express both “M1-like” and “M2-like” signatures concurrently. To determine whether this is the case for our data single-cell sequencing would be necessary. Interestingly, the literature does support the presence of simultaneous activation of pathways categorized as underlying opposing phenotypes and functions. For example, individual monocytes/macrophages demonstrated enrichment of both “M1-like” (IFN-γ and LPS) and “M2-like” (IL-4) signatures following traumatic brain injury (60). However, the degree to which individual cells can be activated toward competing polarization states remains to be established.

To further focus on signaling pathways that may be driving the IFN-γ-related shift in monocyte phenotype in our data we carried out transcription factor enrichment analysis (TFEA) on the *leading-edge subset genes* underlying the IFN-γ signature enrichments. For all group comparisons IRF8 and IRF1 were the primary transcription factors indicated as driving enrichment of these subsets of genes. To address transcriptional control of the overall shift in monocyte phenotype with changing illness duration we carried out an additional analysis using a more unbiased approach, removing the constraints of restricting the analysis to the IFN-γ *leading-edge subset genes*. Including all genes in the medium vs. late illness duration comparison and the entire transcription factor enrichment database, GSEA again highlighted IRF8 and IRF1 as the primary signaling components driving the shift, though this result did not withstand FDR correction. IRF8 and IRF1 are transcription factors that interact with STAT1 following IFN-γ stimulation and are vital for the activation of transcriptional programs underlying both myeloid cell development and the coordination of a wide range of immune and proinflammatory cellular functions (61). They have also been associated with an “M1-like” phenotypic skew (62). The regulation of myeloid cell transcriptional programs as well as effects on chromatin structure under stimulated and basal conditions have been termed the IRF8/IRF1 regulome. Our findings suggest that alterations to activity of the IRF8/IRF1 regulome may underlie the IFN-γ signature changes and drive the monocyte phenotype shifts observed in individuals with psychosis.

The functional implications of these shifts in circulating monocytes for pathophysiology in psychosis remains to be established. It is increasingly clear that peripherally derived cells are present in and act at the brain borders under steady state conditions in addition to increased infiltration in the context of disease (3, 26, 63). Interestingly, data indicate that changes related to immune gene expression in schizophrenia post-mortem brain may be at least partly due to the presence of circulating monocytes in vessels (64). Additionally, activation of the IRF8/IRF1 regulome is associated with neuroinflammation in models of inflammatory disorders whereas loss-of-function mutants for these transcription factors can be protective (61). While increased infiltration of monocytes to the brain has historically been assumed detrimental, and indeed these cells have been demonstrated to contribute to neuroinflammation under many conditions, data suggest that peripherally-derived monocytes skewed toward an M2-like phenotype can also serve neuroprotective and reparative functions (65–67). Finally, in other brain disorders with an immune component, such as Alzheimer's disease, shifts in myeloid phenotype have also been observed with illness progression (26). As here, the beneficial vs. protective functional significance is still not fully elucidated.

The main limitation of this study, as with many genome-wide sequencing investigations, is the sample size. While the sample was intentionally matched for key demographic characteristics, there remain a number of possible confounds such as co-morbidities and associated medications impacting immune function that may be better controlled for within a larger sample. Though no clear differences were found between the groups for variables such as BMI, nicotine use and immune-modulatory medications such as statins and metformin, these variables may well be moderating factors. Additionally, studies indicate that antipsychotic drugs, which all but one participant with schizophrenia were being treated with, are themselves immune-modifying and future work should therefore assess the effects of treatment on monocyte phenotype. While CPZE calculated for the participants treated with oral antipsychotics did not differ between illness duration groups, it may be that long-term antipsychotic treatment contributes to shifts in immune signatures in participants with schizophrenia with cumulative effects over the course of illness. Indeed, we have previously shown that antipsychotics can influence the assembly of promoter chromatin and increase immune gene expression (68, 69).

There are several caveats to interpretation of the exploratory analysis based on illness duration that we would like to emphasize. One of the most important points is that this sample is one of participants with chronic schizophrenia with a mean duration of 32 years. Thus, all participants, even those in the “medium” illness duration group, have had symptoms of schizophrenia for a number of years. This sample of participants with chronic but stable symptoms allowed us to exclude illness acuity as a potential confound, which we have previously found to associate with shifts in the IFN-γ signature. Later years of illness generally see fewer radical fluctuations in psychopathology and treatment, including treatment side effects. However, by examining this narrow window we are unable to make inferences regarding monocyte phenotype across the entire course of illness. Future work should include comparisons based on acuity as well as first-episode/early vs. chronic illness. Additionally, the sample size is very small (7 participants in each subgroup) and therefore the results should be interpreted with caution. While one of the advantages in the illness duration subgroup analysis was that the groups were matched for age, controlling for the effects of age on immune function, this resulted in a difference in age of onset between the two subgroups. Because age of onset may itself be associated with differences in illness, future work should control for both age and age of onset in a larger sample.

Overall, our results provide a significant advance toward understanding the complexity of immune changes in chronic schizophrenia, demonstrating enrichment of pro- and anti-inflammatory signatures in circulating monocytes. The results of the exploratory subsample analysis further support the importance of considering illness stage as a variable when studying immune function and suggest a phenotypic shift in monocytes in the later stages of chronic schizophrenia. These findings may have important ramifications for understanding the role of immune cells in psychosis pathophysiology as well as in the consideration of immune targeting treatment strategies for schizophrenia. For example, clinical trials for immunomodulatory drugs in schizophrenia may benefit from stratifying participants based on current immune measures and illness stage. Additionally, transcriptional signatures have potential utility as outcome measures in determining the effects of treatment on immune function.

## Data Availability Statement

The datasets presented in this article are not readily available because of ethical issue with sharing the data in that signed consent forms did not inform participants that their data would be shared (identified or deidentified) to a public domain. Requests to access the datasets should be directed to Rajiv P. Sharma.

## Ethics Statement

The studies involving human participants were reviewed and approved by Institutional Review Board of the University of Illinois at Chicago. The patients/participants provided their written informed consent to participate in this study.

## Author Contributions

RS, CR, KC, and JM contributed to study design. RS and CR were responsible for participant recruitment and sample collection. JM and BF processed participant samples. JM carried out statistical and bioinformatic analyses and drafted the manuscript, with assistance from CR and RS. The manuscript was edited and approved by all authors.

## Conflict of Interest

The authors declare that the research was conducted in the absence of any commercial or financial relationships that could be construed as a potential conflict of interest.
