# Supplementary material for: Monocyte Transcriptional Profiling Highlights a Shift in Immune Signatures Over the Course of Illness in Schizophrenia
Source: Front Psychiatry. 2021 May 14;12:649494. doi: 10.3389/fpsyt.2021.649494 (PMC8160367; doi:10.3389/fpsyt.2021.649494)
Supplement: Supplementary file 1 [file Table_1.DOCX]

Supplementary Table 1. GSEA carried out using the MSigDB 50 hallmark gene sets.

Schizophrenia compared to controls:

| NAME | SIZE | ES | NES | NOM p-val | FDR q-val |
| --- | --- | --- | --- | --- | --- |
| HALLMARK_INTERFERON_ALPHA_RESPONSE | 84 | 0.484 | 2.284 | 0.000 | 0.000 |
| HALLMARK_INTERFERON_GAMMA_RESPONSE | 173 | 0.388 | 2.087 | 0.000 | 0.000 |
| HALLMARK_OXIDATIVE_PHOSPHORYLATION | 195 | 0.381 | 2.045 | 0.000 | 0.001 |
| HALLMARK_COAGULATION | 60 | 0.419 | 1.850 | 0.000 | 0.003 |
| HALLMARK_ADIPOGENESIS | 155 | 0.347 | 1.807 | 0.000 | 0.006 |
| HALLMARK_REACTIVE_OXIGEN_SPECIES_PATHWAY | 44 | 0.400 | 1.684 | 0.003 | 0.017 |
| HALLMARK_XENOBIOTIC_METABOLISM | 124 | 0.324 | 1.651 | 0.000 | 0.019 |
| HALLMARK_COMPLEMENT | 142 | 0.304 | 1.561 | 0.000 | 0.029 |
| HALLMARK_EPITHELIAL_MESENCHYMAL_TRANSITION | 78 | 0.330 | 1.550 | 0.005 | 0.031 |
| HALLMARK_MYOGENESIS | 96 | 0.303 | 1.493 | 0.005 | 0.043 |
| HALLMARK_HEME_METABOLISM | 150 | 0.279 | 1.456 | 0.006 | 0.053 |
| HALLMARK_BILE_ACID_METABOLISM | 64 | 0.322 | 1.436 | 0.029 | 0.055 |
| HALLMARK_HYPOXIA | 130 | 0.283 | 1.428 | 0.005 | 0.054 |
| HALLMARK_KRAS_SIGNALING_UP | 110 | 0.288 | 1.424 | 0.005 | 0.051 |
| HALLMARK_PEROXISOME | 77 | 0.299 | 1.407 | 0.020 | 0.055 |
| HALLMARK_P53_PATHWAY | 159 | 0.264 | 1.376 | 0.007 | 0.067 |
| HALLMARK_INFLAMMATORY_RESPONSE | 131 | 0.271 | 1.358 | 0.006 | 0.071 |
| HALLMARK_GLYCOLYSIS | 140 | 0.255 | 1.324 | 0.031 | 0.085 |
| HALLMARK_FATTY_ACID_METABOLISM | 123 | 0.255 | 1.307 | 0.041 | 0.094 |
| HALLMARK_APOPTOSIS | 128 | 0.238 | 1.215 | 0.124 | 0.174 |
| HALLMARK_ANDROGEN_RESPONSE | 76 | 0.258 | 1.205 | 0.101 | 0.176 |
| HALLMARK_APICAL_SURFACE | 18 | 0.362 | 1.191 | 0.207 | 0.186 |
| HALLMARK_MTORC1_SIGNALING | 178 | 0.216 | 1.159 | 0.103 | 0.220 |
| HALLMARK_PROTEIN_SECRETION | 88 | 0.213 | 1.010 | 0.414 | 0.517 |
| HALLMARK_CHOLESTEROL_HOMEOSTASIS | 60 | 0.210 | 0.922 | 0.620 | 0.746 |
| HALLMARK_DNA_REPAIR | 132 | 0.174 | 0.916 | 0.711 | 0.734 |
| HALLMARK_IL2_STAT5_SIGNALING | 143 | 0.167 | 0.858 | 0.844 | 0.848 |
| HALLMARK_IL6_JAK_STAT3_SIGNALING | 65 | 0.188 | 0.848 | 0.824 | 0.840 |
| HALLMARK_TNFA_SIGNALING_VIA_NFKB | 162 | 0.154 | 0.815 | 0.940 | 0.866 |

Medium illness duration compared to controls:

| NAME | SIZE | ES | NES | NOM p-val | FDR q-val |
| --- | --- | --- | --- | --- | --- |
| HALLMARK_OXIDATIVE_PHOSPHORYLATION | 195 | 0.420 | 2.297 | 0.000 | 0.000 |
| HALLMARK_HYPOXIA | 130 | 0.355 | 1.819 | 0.000 | 0.018 |
| HALLMARK_ADIPOGENESIS | 155 | 0.314 | 1.666 | 0.000 | 0.046 |
| HALLMARK_REACTIVE_OXIGEN_SPECIES_PATHWAY | 44 | 0.348 | 1.462 | 0.023 | 0.131 |
| HALLMARK_KRAS_SIGNALING_UP | 110 | 0.290 | 1.436 | 0.000 | 0.123 |
| HALLMARK_COAGULATION | 60 | 0.305 | 1.400 | 0.027 | 0.140 |
| HALLMARK_GLYCOLYSIS | 140 | 0.240 | 1.277 | 0.054 | 0.279 |
| HALLMARK_MYOGENESIS | 96 | 0.257 | 1.259 | 0.066 | 0.274 |
| HALLMARK_TNFA_SIGNALING_VIA_NFKB | 162 | 0.230 | 1.228 | 0.085 | 0.301 |
| HALLMARK_P53_PATHWAY | 159 | 0.226 | 1.216 | 0.092 | 0.296 |
| HALLMARK_PEROXISOME | 77 | 0.254 | 1.202 | 0.120 | 0.296 |
| HALLMARK_EPITHELIAL_MESENCHYMAL_TRANSITION | 78 | 0.250 | 1.183 | 0.153 | 0.308 |
| HALLMARK_BILE_ACID_METABOLISM | 64 | 0.240 | 1.104 | 0.232 | 0.480 |
| HALLMARK_HEME_METABOLISM | 150 | 0.201 | 1.095 | 0.220 | 0.475 |
| HALLMARK_APOPTOSIS | 128 | 0.211 | 1.075 | 0.229 | 0.509 |
| HALLMARK_INTERFERON_GAMMA_RESPONSE | 173 | 0.201 | 1.059 | 0.244 | 0.520 |
| HALLMARK_UV_RESPONSE_UP | 113 | 0.205 | 1.039 | 0.347 | 0.553 |
| HALLMARK_MTORC1_SIGNALING | 178 | 0.188 | 1.016 | 0.450 | 0.600 |
| HALLMARK_FATTY_ACID_METABOLISM | 123 | 0.195 | 0.975 | 0.513 | 0.708 |
| HALLMARK_APICAL_SURFACE | 18 | 0.289 | 0.964 | 0.483 | 0.706 |
| HALLMARK_MYC_TARGETS_V1 | 192 | 0.175 | 0.959 | 0.610 | 0.688 |
| HALLMARK_TGF_BETA_SIGNALING | 44 | 0.226 | 0.931 | 0.600 | 0.741 |
| HALLMARK_INFLAMMATORY_RESPONSE | 131 | 0.177 | 0.906 | 0.742 | 0.777 |
| HALLMARK_CHOLESTEROL_HOMEOSTASIS | 60 | 0.195 | 0.882 | 0.751 | 0.806 |
| HALLMARK_ANDROGEN_RESPONSE | 76 | 0.176 | 0.830 | 0.847 | 0.873 |
| HALLMARK_SPERMATOGENESIS | 51 | 0.164 | 0.705 | 0.932 | 0.964 |

Long illness duration compared to controls:

| NAME | SIZE | ES | NES | NOM p-val | FDR q-val |
| --- | --- | --- | --- | --- | --- |
| HALLMARK_INTERFERON_ALPHA_RESPONSE | 84 | 0.587 | 2.709 | 0.000 | 0.000 |
| HALLMARK_INTERFERON_GAMMA_RESPONSE | 173 | 0.448 | 2.346 | 0.000 | 0.000 |
| HALLMARK_COAGULATION | 60 | 0.432 | 1.857 | 0.000 | 0.004 |
| HALLMARK_XENOBIOTIC_METABOLISM | 124 | 0.349 | 1.727 | 0.000 | 0.015 |
| HALLMARK_COMPLEMENT | 142 | 0.320 | 1.613 | 0.000 | 0.035 |
| HALLMARK_EPITHELIAL_MESENCHYMAL_TRANSITION | 78 | 0.315 | 1.425 | 0.025 | 0.113 |
| HALLMARK_BILE_ACID_METABOLISM | 64 | 0.324 | 1.418 | 0.030 | 0.100 |
| HALLMARK_P53_PATHWAY | 159 | 0.273 | 1.389 | 0.000 | 0.108 |
| HALLMARK_HEME_METABOLISM | 150 | 0.257 | 1.304 | 0.053 | 0.193 |
| HALLMARK_PEROXISOME | 77 | 0.282 | 1.290 | 0.072 | 0.191 |
| HALLMARK_REACTIVE_OXIGEN_SPECIES_PATHWAY | 44 | 0.314 | 1.267 | 0.123 | 0.200 |
| HALLMARK_INFLAMMATORY_RESPONSE | 131 | 0.249 | 1.259 | 0.042 | 0.193 |
| HALLMARK_DNA_REPAIR | 132 | 0.240 | 1.185 | 0.094 | 0.292 |
| HALLMARK_OXIDATIVE_PHOSPHORYLATION | 195 | 0.222 | 1.185 | 0.034 | 0.272 |
| HALLMARK_GLYCOLYSIS | 140 | 0.230 | 1.167 | 0.122 | 0.288 |
| HALLMARK_MYOGENESIS | 96 | 0.234 | 1.108 | 0.217 | 0.398 |
| HALLMARK_ADIPOGENESIS | 155 | 0.214 | 1.103 | 0.214 | 0.388 |
| HALLMARK_HYPOXIA | 130 | 0.227 | 1.097 | 0.260 | 0.380 |
| HALLMARK_MTORC1_SIGNALING | 178 | 0.199 | 1.042 | 0.350 | 0.509 |
| HALLMARK_KRAS_SIGNALING_UP | 110 | 0.207 | 1.013 | 0.413 | 0.567 |
| HALLMARK_FATTY_ACID_METABOLISM | 123 | 0.197 | 0.982 | 0.505 | 0.641 |
| HALLMARK_CHOLESTEROL_HOMEOSTASIS | 60 | 0.216 | 0.943 | 0.575 | 0.732 |
| HALLMARK_APICAL_SURFACE | 18 | 0.290 | 0.941 | 0.557 | 0.705 |
| HALLMARK_PROTEIN_SECRETION | 88 | 0.200 | 0.935 | 0.620 | 0.694 |
| HALLMARK_ANDROGEN_RESPONSE | 76 | 0.207 | 0.932 | 0.619 | 0.674 |
| HALLMARK_IL6_JAK_STAT3_SIGNALING | 65 | 0.206 | 0.911 | 0.638 | 0.700 |
| HALLMARK_APOPTOSIS | 128 | 0.175 | 0.880 | 0.805 | 0.744 |

Medium illness duration compared to long illness duration:

| NAME | SIZE | ES | NES | NOM p-val | FDR q-val |
| --- | --- | --- | --- | --- | --- |
| HALLMARK_TNFA_SIGNALING_VIA_NFKB | 162 | 0.397393 | 1.986 | 0.000 | 0.003 |
| HALLMARK_HYPOXIA | 130 | 0.319408 | 1.546 | 0.000 | 0.092 |
| HALLMARK_OXIDATIVE_PHOSPHORYLATION | 195 | 0.29382 | 1.503 | 0.003 | 0.084 |
| HALLMARK_MYC_TARGETS_V1 | 192 | 0.274846 | 1.402 | 0.006 | 0.153 |
| HALLMARK_TGF_BETA_SIGNALING | 44 | 0.34018 | 1.295 | 0.094 | 0.275 |
| HALLMARK_UV_RESPONSE_UP | 113 | 0.270583 | 1.274 | 0.057 | 0.263 |
| HALLMARK_SPERMATOGENESIS | 51 | 0.308909 | 1.250 | 0.128 | 0.274 |
| HALLMARK_MYOGENESIS | 96 | 0.260833 | 1.183 | 0.134 | 0.382 |
| HALLMARK_KRAS_SIGNALING_UP | 110 | 0.231664 | 1.076 | 0.299 | 0.662 |
| HALLMARK_APOPTOSIS | 128 | 0.213083 | 1.015 | 0.401 | 0.836 |
| HALLMARK_ADIPOGENESIS | 155 | 0.204459 | 1.003 | 0.415 | 0.810 |
| HALLMARK_GLYCOLYSIS | 140 | 0.189461 | 0.926 | 0.686 | 1.000 |
| HALLMARK_INFLAMMATORY_RESPONSE | 131 | 0.190641 | 0.917 | 0.669 | 1.000 |
| HALLMARK_ALLOGRAFT_REJECTION | 145 | 0.188067 | 0.914 | 0.649 | 0.942 |
| HALLMARK_UNFOLDED_PROTEIN_RESPONSE | 103 | 0.194241 | 0.896 | 0.698 | 0.933 |
| HALLMARK_MYC_TARGETS_V2 | 54 | 0.221116 | 0.889 | 0.678 | 0.894 |
| HALLMARK_PI3K_AKT_MTOR_SIGNALING | 87 | 0.186989 | 0.842 | 0.803 | 0.960 |
| HALLMARK_BILE_ACID_METABOLISM | 64 | 0.177687 | 0.759 | 0.923 | 1.000 |
| HALLMARK_ANGIOGENESIS | 17 | 0.244406 | 0.748 | 0.809 | 0.989 |
| HALLMARK_CHOLESTEROL_HOMEOSTASIS | 60 | 0.143213 | 0.604 | 0.987 | 0.993 |
