# Supplementary material for: Monocyte Transcriptional Profiling Highlights a Shift in Immune Signatures Over the Course of Illness in Schizophrenia
Source: Front Psychiatry. 2021 May 14;12:649494. doi: 10.3389/fpsyt.2021.649494 (PMC8160367; doi:10.3389/fpsyt.2021.649494)
Supplement: Supplementary file 2 [file Table_2.DOCX]

Supplementary Table 2. GSEA carried out using illness duration as a continuous phenotype.

Using compiled gene sets

| NAME | SIZE | ES | NES | NOM p-val | FDR q-val |
| --- | --- | --- | --- | --- | --- |
| IFN-γ Signature | 434 | 0.358488 | 1.387926 | 0 | 0.1254 |
| IFN-α Signature | 147 | 0.366034 | 1.355223 | 0.01608 | 0.090533 |
| LPS Acute Signature | 277 | 0.298466 | 1.145816 | 0.131 | 0.437579 |
| GC Acute Signature | 63 | 0.269948 | 0.933379 | 0.598947 | 1 |
| Chronic Stress Signature | 149 | 0.242284 | 0.898773 | 0.738167 | 1 |
| ET Signature | 48 | 0.265878 | 0.886666 | 0.660657 | 0.870336 |
| IL-4 Signature | 107 | 0.219705 | 0.803355 | 0.848638 | 0.871769 |

Using MSigDB Hallmark 50 gene sets.

| NAME | SIZE | ES | NES | NOM p-val | FDR q-val |
| --- | --- | --- | --- | --- | --- |
| HALLMARK_INTERFERON_ALPHA_RESPONSE | 85 | 0.506 | 1.800 | 0.000 | 0.002 |
| HALLMARK_PROTEIN_SECRETION | 87 | 0.500 | 1.780 | 0.000 | 0.001 |
| HALLMARK_MTORC1_SIGNALING | 176 | 0.416 | 1.563 | 0.000 | 0.043 |
| HALLMARK_INTERFERON_GAMMA_RESPONSE | 173 | 0.412 | 1.546 | 0.000 | 0.040 |
| HALLMARK_XENOBIOTIC_METABOLISM | 123 | 0.416 | 1.528 | 0.003 | 0.042 |
| HALLMARK_COMPLEMENT | 146 | 0.396 | 1.471 | 0.002 | 0.075 |
| HALLMARK_PI3K_AKT_MTOR_SIGNALING | 88 | 0.397 | 1.422 | 0.014 | 0.113 |
| HALLMARK_G2M_CHECKPOINT | 150 | 0.371 | 1.384 | 0.010 | 0.153 |
| HALLMARK_EPITHELIAL_MESENCHYMAL_TRANSITION | 81 | 0.392 | 1.384 | 0.016 | 0.136 |
| HALLMARK_GLYCOLYSIS | 140 | 0.370 | 1.375 | 0.011 | 0.136 |
| HALLMARK_COAGULATION | 61 | 0.392 | 1.369 | 0.035 | 0.134 |
| HALLMARK_HEME_METABOLISM | 147 | 0.363 | 1.357 | 0.014 | 0.140 |
| HALLMARK_HEDGEHOG_SIGNALING | 17 | 0.491 | 1.342 | 0.093 | 0.150 |
| HALLMARK_NOTCH_SIGNALING | 24 | 0.449 | 1.320 | 0.104 | 0.174 |
| HALLMARK_P53_PATHWAY | 157 | 0.354 | 1.318 | 0.016 | 0.165 |
| HALLMARK_E2F_TARGETS | 160 | 0.351 | 1.306 | 0.029 | 0.175 |
| HALLMARK_FATTY_ACID_METABOLISM | 123 | 0.351 | 1.304 | 0.030 | 0.167 |
| HALLMARK_ANDROGEN_RESPONSE | 74 | 0.372 | 1.302 | 0.076 | 0.161 |
| HALLMARK_DNA_REPAIR | 139 | 0.346 | 1.281 | 0.052 | 0.188 |
| HALLMARK_UV_RESPONSE_DN | 93 | 0.346 | 1.255 | 0.090 | 0.226 |
| HALLMARK_CHOLESTEROL_HOMEOSTASIS | 60 | 0.353 | 1.214 | 0.160 | 0.308 |
| HALLMARK_IL2_STAT5_SIGNALING | 144 | 0.311 | 1.166 | 0.152 | 0.426 |
| HALLMARK_PEROXISOME | 78 | 0.326 | 1.165 | 0.195 | 0.412 |
| HALLMARK_MITOTIC_SPINDLE | 170 | 0.305 | 1.143 | 0.164 | 0.460 |
| HALLMARK_APOPTOSIS | 128 | 0.310 | 1.141 | 0.220 | 0.450 |
| HALLMARK_BILE_ACID_METABOLISM | 64 | 0.324 | 1.137 | 0.264 | 0.443 |
| HALLMARK_ADIPOGENESIS | 160 | 0.303 | 1.134 | 0.202 | 0.437 |
| HALLMARK_APICAL_SURFACE | 19 | 0.404 | 1.119 | 0.329 | 0.465 |
| HALLMARK_ESTROGEN_RESPONSE_LATE | 100 | 0.296 | 1.081 | 0.345 | 0.564 |
| HALLMARK_KRAS_SIGNALING_UP | 113 | 0.290 | 1.066 | 0.354 | 0.593 |
| HALLMARK_KRAS_SIGNALING_DN | 52 | 0.310 | 1.038 | 0.433 | 0.663 |
| HALLMARK_INFLAMMATORY_RESPONSE | 134 | 0.278 | 1.034 | 0.415 | 0.656 |
| HALLMARK_MYC_TARGETS_V2 | 54 | 0.304 | 1.021 | 0.455 | 0.673 |
| HALLMARK_UNFOLDED_PROTEIN_RESPONSE | 102 | 0.278 | 1.015 | 0.467 | 0.673 |
| HALLMARK_OXIDATIVE_PHOSPHORYLATION | 182 | 0.262 | 0.983 | 0.553 | 0.746 |
| HALLMARK_IL6_JAK_STAT3_SIGNALING | 65 | 0.280 | 0.973 | 0.552 | 0.753 |
| HALLMARK_MYOGENESIS | 96 | 0.264 | 0.954 | 0.591 | 0.786 |
| HALLMARK_MYC_TARGETS_V1 | 191 | 0.250 | 0.945 | 0.638 | 0.788 |
| HALLMARK_REACTIVE_OXYGEN_SPECIES_PATHWAY | 47 | 0.281 | 0.941 | 0.586 | 0.779 |
| HALLMARK_ANGIOGENESIS | 17 | 0.341 | 0.936 | 0.555 | 0.772 |
| HALLMARK_UV_RESPONSE_UP | 114 | 0.257 | 0.933 | 0.635 | 0.760 |
| HALLMARK_APICAL_JUNCTION | 109 | 0.239 | 0.874 | 0.746 | 0.864 |
| HALLMARK_HYPOXIA | 132 | 0.237 | 0.870 | 0.757 | 0.853 |
| HALLMARK_WNT_BETA_CATENIN_SIGNALING | 28 | 0.274 | 0.846 | 0.710 | 0.874 |
| HALLMARK_SPERMATOGENESIS | 50 | 0.231 | 0.781 | 0.823 | 0.947 |
| HALLMARK_ESTROGEN_RESPONSE_EARLY | 109 | 0.201 | 0.731 | 0.926 | 0.977 |
| HALLMARK_TGF_BETA_SIGNALING | 44 | 0.219 | 0.728 | 0.879 | 0.959 |
| HALLMARK_ALLOGRAFT_REJECTION | 140 | 0.190 | 0.708 | 0.958 | 0.954 |
| HALLMARK_TNFA_SIGNALING_VIA_NFKB | 162 | 0.187 | 0.703 | 0.966 | 0.938 |
